# Laparoscopic management of Rhabdomyosarcoma of common Bile duct, Case report

**DOI:** 10.1016/j.amsu.2020.09.023

**Published:** 2020-09-20

**Authors:** T.M. Al Quran, L.A. Rousan, A.M. Aljaafreh, Z.A. Bataineh

**Affiliations:** a Department of Public Health/Community Medicine and Family Medicine, Jordan; b Department of Diagnostic and Interventional Radiology and Nuclear Medicine, Jordan; c Department of General and Pediatric Surgery, Jordan

**Keywords:** Rhabdomyosarcoma, Choledochal cyst, Common bile duct, Laparoscope, Case report

## Abstract

**Introduction:**

Embryonal Rhabdomyosarcoma (ERMS) is a malignant soft tissue musculoskeletal tumor which constitutes about 0.06% of all malignancies affecting children. Biliary tract ERMS is still rare, though it is considered the most common cause of malignant obstructive jaundice in children.

**Case presentation:**

A report of a 2-year-old boy, who was presented with recurrent episodes of scleral icterus of three months duration, is added to the related literature. His labs went with obstructive jaundice and the radiological investigations were consistent with a diagnosis of choledochal cyst. The found mass was suspected to be an ERMS of common bile duct and turned out to be so by the histopathology. He was managed totally by laparoscope, both excision and hepaticojejunostomy reconstruction, which is an extremely uncommon entity.

**Conclusion:**

Common Bile Duct Rhabdomyosarcoma is rare and diagnosis at this anatomical site is difficult. Our case highlights the feasibility of laparoscopic resection and hepaticojejunostomy reconstruction with very good results at 16-month follow up and parents' gratitude as well.

## Highlights

• Rhabdomyosarcoma (RMS) is a rare malignant tumor affecting children.
• RMS is worth considering when dealing with a choledochal cyst.
• Surgical approaches reported for biliary RMS were laparotomies.
• Laparoscopic management is feasible for biliary RMS.
• Our case is the least invasive surgical approach reported up to date.

## 1 Introduction

Rhabdomyosarcoma (RMS) is the most common soft tissue tumor in children. However, they are rare as they constitute about 3% of all malignancies affecting pediatrics [1], with median age of 3.5 years and slight male predominance [[2], [3], [4], [5]]. RMS is divided into five major subtypes; Embryonal subtype is considered the most common one [6]. The common sites in pediatric age group are head, neck, genitourinary, and retroperitoneal [7]. Although common Bile duct (CBD) affection is rare, it is considered to be the most common cause of malignant obstructive jaundice in children [3,5]. Biliary tract RMS was first described by Wilks and Moxon in 18,75 [8].

ERMS looks like the radiological and clinical features of Choledochal Cyst (CC); diagnosis is usually made at surgery [[2], [3], [4], [5]]. The management of RMS depends on risk stratification and involves one or a combination of surgical resection, chemotherapy and radiotherapy [9]. Minimally invasive surgery (MIS) in pediatric age group has been expanded to the extent that complex procedures like laparoscopic choledochal cyst surgery can be conducted with safety and outcomes similar to open approach and with the advantage of minimal scarring [10,11]. However, surgical approaches reported for biliary RMS were laparotomies except one case, which resection was done by robot and converted to open approach for Roux-en-Y Hepaticojejunostomy (RYHJ) reconstruction [2].

We are reporting a case of a 2-year old boy presented with attacks of jaundice because of CBD ERMS that was diagnosed initially as CC, managed totally with laparoscopic excision and Roux-en-Y Hepaticojejunostomy (RYHJ).

This work has been reported in line with the Surgical Case Report (SCARE) criteria [12].

## 2 Case presentation

A 2-year-old boy, not known to have any medical or congenital diseases, referred to our tertiary Hospital by his family physician due to recurrent attacks of scleral icterus for 3 months, almost with same intensity, with no fever, abdominal pain, and change in stool nor urine color. He has no history of allergy and his parents denied regular or recent drug intake. The family and psychosocial history were unremarkable. His physical examination was unremarkable, except yellowish sclera bilaterally. Laboratory tests revealed mild elevation of white blood cells, elevated levels of total bilirubin, direct bilirubin, and alkaline phosphatase. Abdominal ultrasound showed fusiform dilatation of the Common Bile Duct with sludge consistent with CC type I, the liver and spleen were homogeneous with no focal lesions and the gall bladder was partially contracted with no stone inside. A diagnosis of CC was further supported by magnetic resonance cholangiopancreatography (MRCP). After informed consent signed by his father, the patient was booked for minimally invasive CC excision with Roux-en Y Hepaticojejunostomy (RYHJ) surgery.

This procedure performed by a consultant pediatric surgeon subspecialized in minimally invasive surgery in pediatric age group at one of reputable centers in Europe, has a good audit of laparoscopic CC excision and Roux-en-Y hepaticojujonostomy reconstruction. The patient was placed in a supine position, an infra-umbilical incision by open Hasson technique used for 10 mm trocar, 30° lens, and Co_2_ pneumoperitoneum was established at a pressure of 8 mmHg and flow of 5 L/min. Another 10 mm port was placed just below the left costal margin medially, two additional 5 mm trocars were inserted in the right lower quadrant and an adequate exposure was achieved by retracting the liver and elevating the gallbladder to allow dissection of the CC and freeing it from the surrounding structures. The CBD was 3 cm fusiform in shape, with the help of camera magnification meticulous dissection and resection of proximal part of common hepatic duct was done, almost up to the confluence of right and left hepatic that permit anastomosis, with small lymph nodes (LN) around, this approach gave us superior field view over open approach. CBD was divided after clip placement distally as close as possible to pancreaticobiliaery junction.

**Fig. 1:**
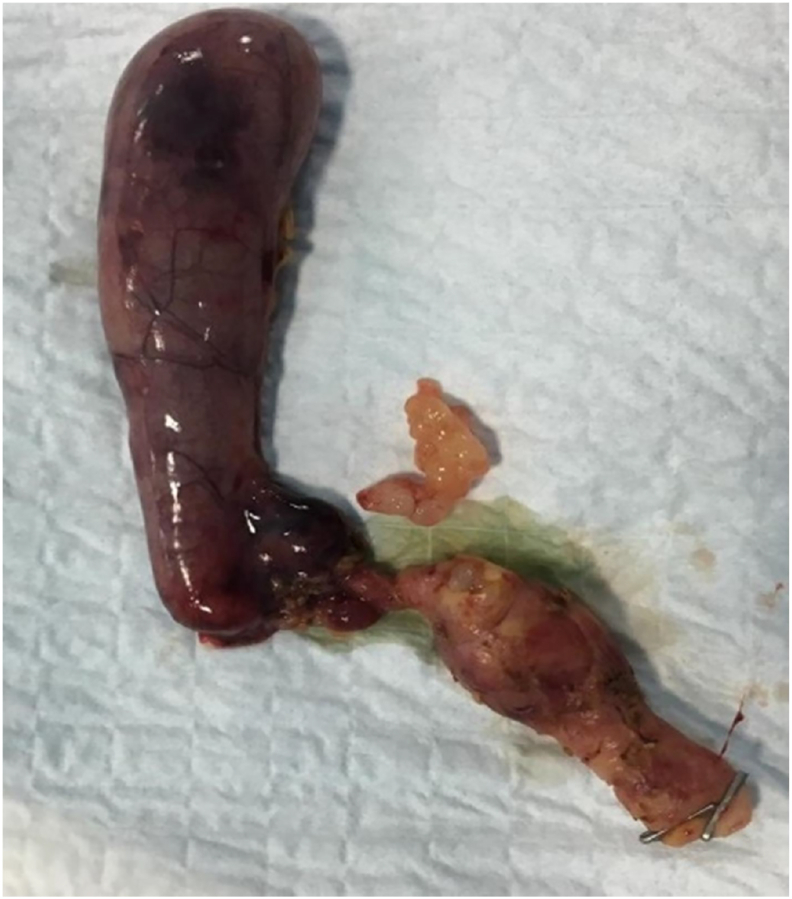
Resected Choledochal Cyst containing grapelike soft tissue specimen.

**Fig. 2:**
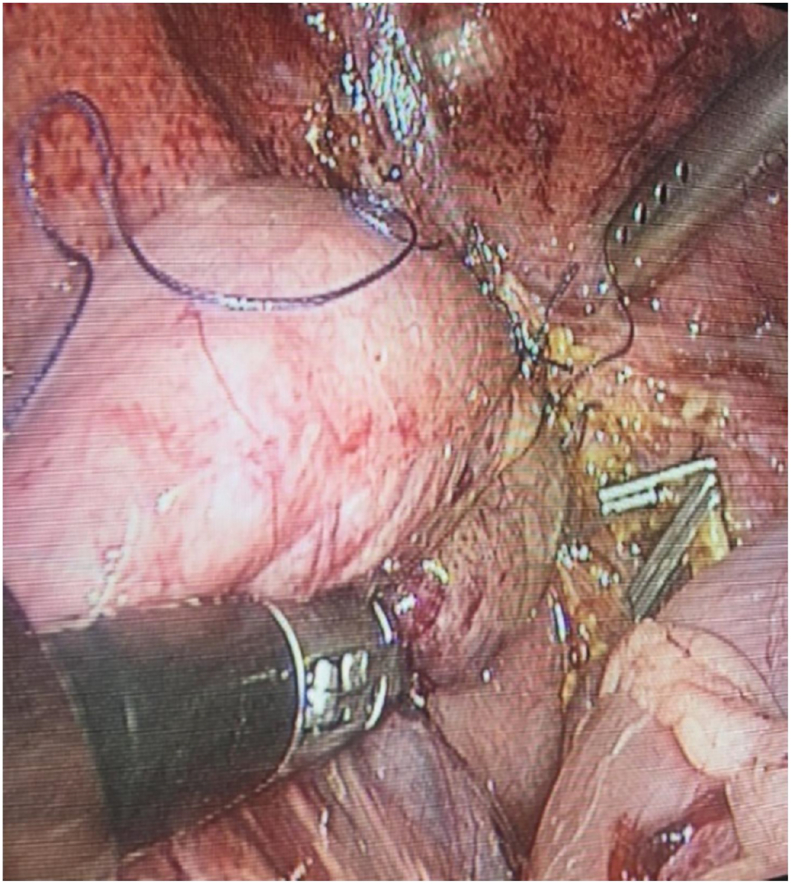
Hepaticojejunostomy reconstruction using laparoscope.

A grapelike soft tissue mass that appeared within the excised part of common hepatic duct lumen made a new diagnostic challenge that raised the possibility of biliary RMS, while the procedure went through same plan; the gall bladder was freed and excised with the sample en-block {Fig. 1}. The specimen retrieved by endobag through the umbilical port after it had been extended up to 3 cm length, this was adequate to exteriorize the jejunum and anastomose the jejunojejunostomy of the Roux-en-Y limb extracorporeally, content returned back to coelomic cavity and pneumoperitoneum re-established again. The biliary tree washed out by normal saline 0.9%, a retro colic tunnel created through which the Roux limb delivered to the porta hepatis, an end to side hepaticojejunostomy accomplished laparoscopically using interrupted 4/0 absorbable sutures {Fig. 2}. A vacuum drain size 14 Fr. was inserted through the lower 5 mm ports to the Morison's pouch and the omentum was placed over the hepaticojejunostomy anastomosis.

**Fig. 3:**
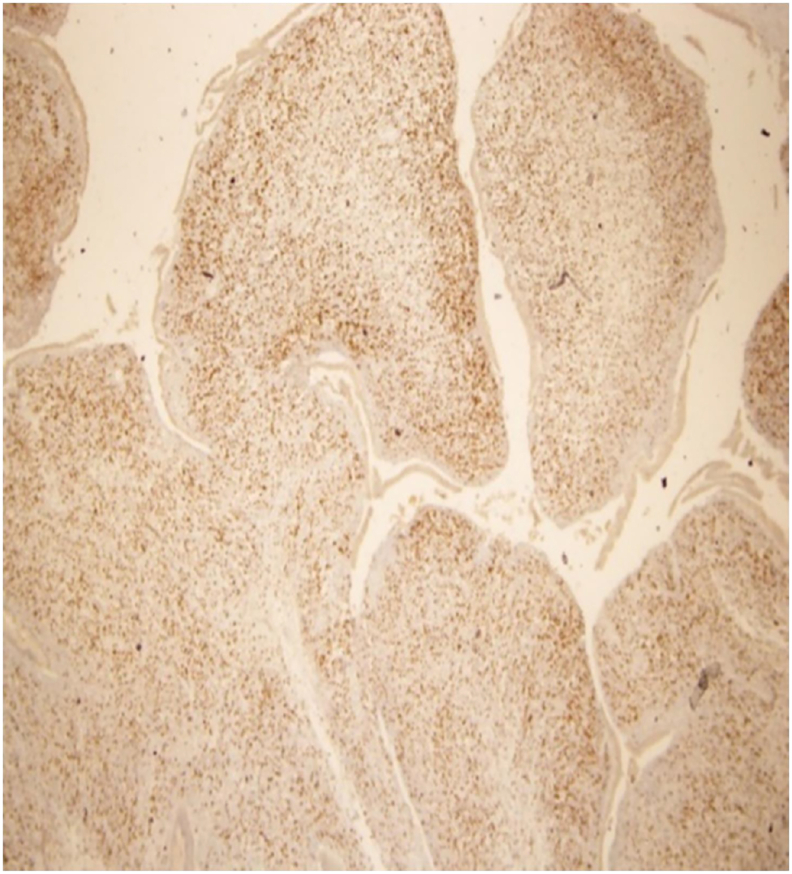
Histopathology: microscopic findings of Embryonal Rhabdomyosarcoma, epithelial surface with underlying condensed layer of primitive spindle cells (Cambium).

On the first post-operative day, the general condition of the patient went uneventful, with 16.6 μmol/L total bilirubin and 14.2 μmol/L direct bilirubin, 1045 IU/L alkaline phosphatase, post-operative course went smooth and feeding began on day five post-operative. The Histopathologist confirmed biliary embryonal RMS tumor with cells at the resection margin and tumor free two Lymph nodes, the tumor cells were positive for Myogenin and Desmin immunostains {Fig. 3}. The patient was discharged home on day six, and re-admitted to oncology team two weeks post-operatively; he tolerated chemotherapy according to Children Oncology Group (COG) protocol for six months. Positron Emission Tomography (PET) scan showed no hypermetabolic masses to suggest neither recurrence nor metastasis. Sixteen months follow up after surgery, the patient has been growing and doing well with no complaints, Though the parents were thinking it was a minor illness, they realized thereafter the real problem and the way of management with excellent prospect. They were advised for follow-up at six months interval.

## 3 Discussion

RMS is a rare malignant tumor affecting children, forming about 2% present at birth [7], it is divided into five major subtypes; Embryonal subtype is considered the most common one (60% of total RMS), and has a better prognosis than other histologic subtypes [6]. CBD affection still rare, but it is the most common cause of malignant obstructive jaundice in children [3,5].

Around one hundred cases of CBD RMS reported in literature [[2], [3], [4],^6^,[13], [14], [15]], the largest series was 25 cases over 25 years [15]. Most of the reported cases were difficult to diagnose and were misdiagnosed as a Choledochal Cyst [[2], [3], [4], [5],^15^], especially in confined tumor with no invasion to the surrounding structures [4]. Radiological evaluation may be deficient to define the lesion due to the consistency of tumor being both solid and cystic [2], ERMS has been well known to mimic radiological and clinical features of CC, diagnosis usually made at surgery [[2], [3], [4], [5]], resection has to be the treatment of choice. Even without preoperative diagnosis of malignancy [2].

The management of RMS is complicated and requires multimodal treatment that includes surgery, chemotherapy, and radiotherapy [9].

Complete removal of the main tumor is the goal whenever possible. In some cases, surgery may be done even if it's clear that all of the cancer can't be removed “functional resection”. As re-operating on the patient due to incomplete resection was accompanied with poorer prognosis, meticulous dissection with good camera magnification i s crucial and offers careful intra-surgical assessment to choose the most suitable intervention in order of resecting the tumor and guarantee optimal outcomes. The safety of laparoscopic CC management and feasibility porta hepatis anastomosis has been proved in most recent reports [10,11]; the incidence of port-site metastasis after undergoing MIS surgery for biliary malignancies is relatively low, thus MIS is indicated [16].

Chemotherapy is needed for every RMS patient, regimens vary by the risk group [16]. Moreover, chemotherapy has been started before the primary surgery to increase the rate of complete resection in some cases [17]. Radiotherapy is accomplished according to the extent of tumor resection [9].

To the best of our knowledge, there is only one report advocate meticulous dissection and excision of CC contains RMS using Robot, while the procedure converted to open for hepaticojejunostomy reconstruction [2]. We are reporting, with the best of our knowledge, a new case of ERMS presented as CC and managed by the least invasive approach up to date, sixteen months following up the patient was with no complaint, radiology free disease and satisfactorily growth, parents express their happiness and gratitude to the outcomes. Our case carries almost all the good prognostic features, and falls under the group I stage I disease according to Intergroup Rhabdomyosarcoma Study Group (IRSG) modified TNM staging [18,19].

## 4 Conclusion

Rhabdomyosarcoma is worth considering when dealing with choledochal cyst. Surgical approaches reported for biliary Rhabdomyosarcoma were laparotomies. Laparoscopic hepaticojejunostomy reconstruction is feasible when facing a grapelike tumor inside the common bile duct. The report of Nakib et al. makes us believe that our case is the least invasive reported one up to date.

## Ethical approval

Ethical approval, IRB and informed consent obtained from patient father had been taken under Ref. 28/127/2019, date October 02, 2019.

## Consent

Written informed consent was obtained from the patient father for publication of this case report and accompanying images. A copy of the written consent is available for review by the Editor-in-Chief of this journal on request.

## Registration of research studies

1. Name of the registry: Research Registry.

2. Unique Identifying number or registration ID: 5870.

3. Hyperlink to your specific registration (must be publicly accessible and will be checked): https://www.researchregistry.com/browse-the-registry#home/

## Guarantor

Thekraiat Majed Al Quran.

## Informed consent

Written informed consent was obtained from the patient father for publication of this case report and accompanying images. A copy of the written consent is available for review by the Editor-in-Chief of this journal on request.

## Sources of funding

None.

## Provenance and peer review

Not commissioned, externally peer reviewed.

## Credit author contribution statement

**Al Quran Tm:** Conceptualization, Writing - original draft, design and, Methodology, Supervision, Resources, collection, Writing - drafting initial manuscript, critical revision the manuscript, Agreed and approved the final draft for submission. **Rousan LA:** Supervision, Writing - original draft, Resources, collection, Critical revision the manuscript, Agreed and approved the final draft for submission. **A.M. Aljaafreh:** Supervision, Writing - review & editing, Resources, collection, Reviewing and editing the manuscript, Agreed and approved the final draft for submission. **Bataineh Za:** Conceptualization, methodology, Resources, Writing - original draft, Writing - review & editing, Supervision, Project Administratio, Writing - original draft, Writing - review & editing, Visualization, Supervision, Project Administration.

## Declaration of competing interest

All authors have NO financial, personal nor conflicts of interest to disclose.

All authors disclose that NO funding to be declared.
